# Behind Enemy Lines: Vital Echocardiographic Data Prior to Ventricular Arrhythmia Ablation

**DOI:** 10.3390/diagnostics12092109

**Published:** 2022-08-31

**Authors:** Silvia Deaconu, Alexandru Deaconu, Gabriela Marascu, Ioana Petre, Radu Vatasescu

**Affiliations:** 1 Cardiology Department, Clinical Emergency Hospital, 014461 Bucharest, Romania; 2 Faculty of Medicine, Carol Davila University of Medicine and Pharmacy, 050474 Bucharest, Romania

**Keywords:** echocardiography, cardiac imaging, ventricular arrhythmia, intracardiac echocardiography

## Abstract

Ventricular arrhythmias (VA) are a major cause of sudden cardiac death (SCD). Echocardiography is the first widely available imaging tool which guides VA management strategies. Along with other invasive and noninvasive imaging techniques, it provides essential information for identification of VA substrate such as differentiation between ischemic and non-ischemic etiology and identification of structural heart disease. Both classic as well as novel echocardiographic techniques such as left ventricular strain measurement and mechanical dispersion assessment provide prognostic information and assist in risk stratification. Furthermore, intracardiac echocardiography may have an adjunctive role for the VA ablation by providing real-time visualization of cardiac structures, continuous monitoring of catheter location and early recognition of procedural complications. This review gathers all relevant information that echocardiography may offer prior to VA ablation procedures.

## 1. Introduction

Ventricular arrhythmias (VA) consist of a large spectrum of ventricular rhythm disturbances ranging from premature ventricular complexes to ventricular tachycardia and fibrillation. VA are a major cause of sudden cardiac death (SCD) and a significant burden on the patient’s quality of life. In the majority of cases, VA are associated with structural heart disease.

Cardiac imaging plays an essential role in revealing the underlying etiology of VA and for SCD risk stratification. Echocardiography is the first readily available, inexpensive and accurate imaging tool used to identify the structural changes associated with VA. It is usually followed by multimodality imaging techniques such as cardiac magnetic resonance (CMR), cardiac computed tomography (CT) and nuclear imaging which bring additional information about the VA substrate. Basic echocardiography measurements such as left ventricle ejection fraction (LVEF) have long been used to assess the risk of SCD and current guidelines still rely on it for the indication of a implantable cardiac defibrillator (ICD) [1].

Substrate ablation is an efficient interventional treatment for VA. For a safe and successful ablation there is a need to understand anatomical VA substrate along with the electric substrate defined by the electroanatomic mapping (EAM). This review will focus on the necessary information provided by the echocardiography prior to the VA ablation procedure.

## 2. Role of Echocardiography in Identification of VA Substrate

### 2.1. VA in Structural Heart Disease

Echocardiography is useful in the diagnosis of structural heart disease [2]. Transthoracic echocardiography (TTE) is a widely available imaging technique which offers valuable information about VA substrate. The main objectives of TTE evaluation are assessment of left ventricle (LV) systolic function with estimation of LVEF, differential diagnosis between ischemic versus nonischemic etiology, assessment of valve function [3], identification of congenital heart disease [3] and different cardiomyopathies [3].

**Figure 1:**
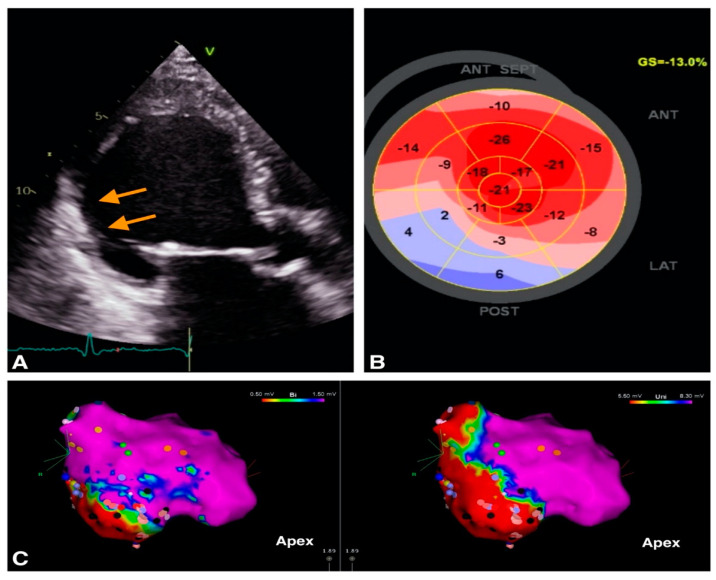
Example of image integration from a patient with VT and old myocardial infarction; (**A**). 2D apical 3 chambers view showing the thin, hyperechogenic inferolateral wall (orange arrows). (**B**). Bull’s eye representation of peak longitudinal strain showing positive values in inferior wall providing a description of the myocardial scar (**C**) Electroanatomic bipolar (left) and unipolar (right) voltage maps in septal view, with an inferior area of low voltage corresponding to the scar identified by echocardiography.

For the evaluation of LV systolic function, LVEF is the main parameter used in VA management. The ESC guidelines recommend performing echocardiography in all patients with suspected or known VA for assessment of LV function and identification of structural substrate [1]. In patients with structural heart disease, a LVEF < 35% estimated by echocardiography is associated with increased risk of VA and SCD [1]. Furthermore, indications for ICDs in primary prevention of SCD rely on LVEF [1]. The LVEF may be estimated using 2D or 3D LV volumes [1]. Furthermore, TTE can identify segmental LV contraction abnormalities and describe myocardial scars which may represent VA substrate (Figure 1).

Transthoracic and transesophageal echocardiography are the routine tools for the diagnosis and severity assessment of underlying valvular heart disease which may contribute to VA occurrence.

Echocardiography plays a significant role in the diagnosis and in management of patients with congenital heart disease and VA. Several studies have shown that right ventricle (RV) and LV dysfunction are risk factors for VA in congenital heart disease patients [2]. It is mandatory to establish the current status of the congenital heart disease and the need of correcting residual complications in order to limit the risk of VA recurrence. A frequent clinical scenario is the operated patient with tetralogy of Fallot with severe residual pulmonary regurgitation and severe dilatation of RV who develops ventricular tachycardia (VT). The treatment of pulmonary stenosis/regurgitation is discussed to promote RV reverse remodeling and maybe decrease the risk of malignant polymorphic VA [4].

A multicenter study performed by Koyak et al. showed that moderate to severe systemic or subpulmonary ventricular dysfunction were associated with a high risk of SCD, especially in adults with transposition of great arteries, Eisenmenger physiology and surgically repaired tetralogy of Fallot [5].

**Figure 2:**
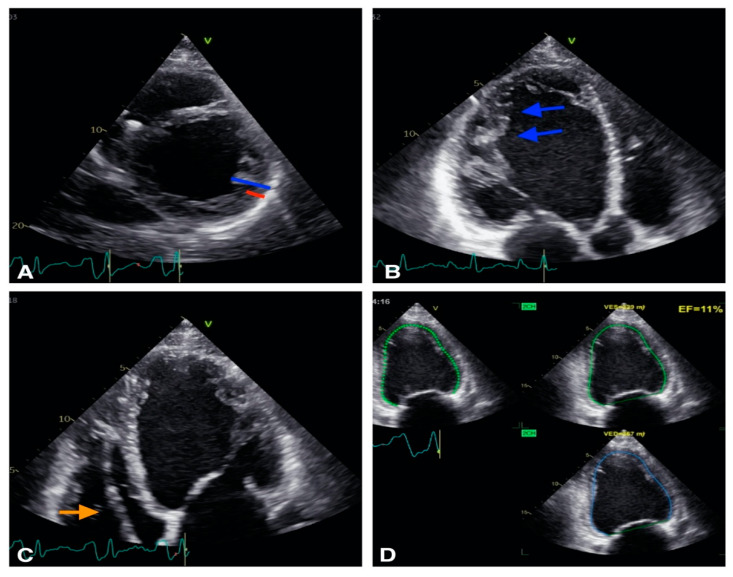
Example of a patient with non-compaction cardiomiopathy. (**A**): 2D short axis view, the non compaction myocardium (blue) is twice thicker than the compacted myocardium (red). (**B**): 2D apical 3 chambers view, trabeculations are visible (blue arrows). (**C**): 2D apical 4 chambers view: defibrillator lead is visibile in right ventricle (orange arrow). (**D**): Biplane EF is calculated at 11% showing severe LV systolic dysfunction.

Finally, echocardiography may diagnose cardiomyopathy as the substrate for VA (Figure 2).

The established echocardiographic criteria for diagnosis of the different cardiomyopathies are summarized in Table 1. However, CMR provides more precise information of VA substrate, as it is able to distinguish between ischemic and non-ischemic etiology and detect subtle structural abnormalities such as myocarditis, sarcoid or amyloid cardiomyopathies [6]. CMR also brings incremental information for the diagnosis of HCM and ARVD [6].

### 2.2. VA in Structurally Normal Hearts

Almost 50% of SCD events occur in patients without structural heart disease [1]. Table 2 summarizes the possible etiology of VA in patients without any obvious structural changes. There is data that “normal” structural hearts associated with VA and channelopathies actually show structural changes. A group by Scheirlynck et al. studied 175 patients with Brugada Syndrome (BrS) compared to 82 controls and found that BrS patients had lower longitudinal strain and more heterogeneous contractions than healthy controls [13]. Furthermore, BrS patients with a history of life-threatening VA had more heterogeneous LV contractions [13]. Therefore, LV mechanical dispersion may be a risk marker in BrS and its evaluation in prospective studies is needed [9]. Studies on RV function also seem to be relevant in BrS patients, in whom subtle contractile RV mechanical abnormalities were demonstrated, including impaired RV longitudinal strain and a greater contraction delay between the lateral and the septal aspect of the RV [14].

Other studies show an overlap between different VA etiology as for BrS and arrhythmogenic cardiomyopathy [15]. The presence of arrhythmogenic cardiomyopathy diagnostic criteria in BrS patients was associated with a trend towards higher arrhythmic risk [15].

Prior to VA ablation, echocardiographic data can be completed with cardiac magnetic resonance (CMR). CMR may be later combined with EAM information. Several studies have confirmed that low endocardial voltage corresponds to the presence of scar tissue as defined by the late gadolinium enhanced CMR [16]. Pre-procedural CMRs have been extremely helpful in localizing scar tissue in the ventricles and have helped to guide and target the arrhythmogenic tissue with catheter ablation [17].

## 3. Echocardiography for Risk Stratification in Patients with VA

Echocardiography plays an important role in SCD risk stratification. Reduced LVEF is associated with a high risk of VA and mortality, but the ability to predict VA is limited [2]. Some of the patients with SCD after myocardial infarction have LVEF > 35% which proves the poor sensitivity of LVEF for risk stratification [2].

Other echocardiographic markers, which are additive to LVEF, are associated with high risk of VA: low global and/or regional strain, low relative wall thickness, worsening mechanical dyssynchrony, high peak strain dispersion (Table 3) [18].

Global wall motion score index (GWMSI) is a prognostic echocardiographic parameter that evaluate wall motion abnormalities and helps to differentiate between ischemic and nonischemic etiology [2]. Mahenthiran et al. showed a predictive value for the composite endpoint of appropriate ICD therapy and all-cause mortality [23]. Restrictive mitral filling pattern and E/E’ ratio are diastolic functional parameters assessed easily by echocardiography, and Bruch et al. suggested that in patients with systolic dysfunction and ICD these parameters were independent predictors of ICD discharge and cardiac death [2].

The evaluation of exercise- or dobutamine-induced wall motion abnormalities could provide additional value to LVEF for risk stratification for VA in patients with coronary artery disease. Also, Elhendy et al. showed that ischemia during stress echocardiography is an independent predictor of death and ICD therapy in patients with coronary heart disease at high risk of arrhythmic death [24].

Relative wall thickness (RWT) is defined as twice the posterior wall thickness divided by the LV diastolic diameter. A study which included patients enrolled in the MADIT-CRT trial demonstrated a powerful predictive value of low RWT for estimating the risk of VA and VA risk or death in either ischemic or nonischemic cardiomyopathy, heart failure and left bundle branch block (LBBB) [2,11].

The reduction of LV global longitudinal strain (GLS) and regional longitudinal myocardial deformation, especially low inferior strain, was associated with the occurrence of VA, irrespective of etiology of cardiomyopathy [18,25,26]. Abnormalities of myocardial deformation or strain can indicate the presence of scar [27]. Furthermore, Trivedi et al. demonstrated that strain abnormalities can quantify the extent of low-voltage scar detected by EAM in ischemic [27] and non-ischemic cardiomyopathy [28]. The results of these studies indicate that performing noninvasive speckle tracking echocardiography may provide valuable information on scar burden and location prior to invasive EAM [28].

Mechanical dispersion is a measure of myocardial deformation heterogeneity and it is regarded as a novel risk parameter of VA, independently of LVEF [2]. It is defined as the standard deviation of time to peak negative strain in all LV myocardial segments [2]. A prospective study by Haugaa et al. conducted upon 569 patients at 40 days after myocardial infarction (MI) show that a combination of mechanical dispersion and global strain may improve the selection of patients after MI for implantable cardioverter defibrillator therapy, particularly in patients with LVEFs > 35% who did not fulfill current ICD indications [29].

In patients with ischemic cardiomyopathy, Mahenthiran et al. showed that GWMSI and right coronary region infero-posterior akinesia identified those at increased risk of events [23]. Ersboll et al. revealed that novel echocardiographic parameters such as GLS and mechanical dispersion were significantly and independently related to SCD or VA after acute MI [25].

In hypertrophic cardiomyopathy (HCM), the most frequent fatal arrhythmia is spontaneous fast monomorphic ventricular tachycardia (VT), whereas non-sustained ventricular tachycardia (NSVT) is often found on ECG monitoring [9]. HCM risk SCD variables include age, family history of SCD, unexplained syncope, NSVT, LV outflow gradient, maximum LV wall thickness, LA diameter [2]. The last three can be easily assessed by echocardiography, but strain parameters may improve risk stratification of VA [2]. Extreme LV hypertrophy defined as maximal wall thickness > 30 mm is associated with higher risk for VA, especially in young patients [30]. Haland et al. showed that worse GLS, more pronounced mechanical dispersion and higher percent of late gadolinium enhancement (%LGE) on CMR were identified in patients with VA, and mechanical dispersion correlated with extent of fibrosis and was an independent risk factor for VA [31].

Echocardiography plays a central role in diagnosis of arrhythmogenic right ventricular cardiomyopathy (ARVC), showing regional RV akinesia, dyskinesia or aneurysm [2]. In patients with early ARVC, larger RV diameter, increased RV mechanical dispersion and reduced LV and RV function are echocardiographic parameters which improve risk stratification of arrhythmic events [32]. Moreover, Mast et al. demonstrated that a prolonged subtricuspid electromechanical interval was an echocardiographic parameter significantly associated with arrhythmogenesis in asymptomatic mutation carriers [33].

In non-compaction cardiomyopathy (NCM) echocardiography is the first-line imaging modality but cannot provide tissue characterization or accurate evaluation of RV function [34]. CMR provides a 3D approach allowing imaging of the entire heart, including both left and right ventricle, with low operator variability or limitations due to patient’s body structure [34] proving the pivotal role of multimodality cardiovascular imaging in the identification and risk stratification of non-compaction cardiomyopathy patients. Left ventricular non compaction (LVNC) is also prevalent in patient with congenital heart disease and might contribute to myocardial dysfunction or arrhythmias [20].

Patients with dilated cardiomyopathy (DCM) are at high risk of SCD and the annual event rate of sustained VA is 4.5% [22]. LVEF < 30–35% was investigated in several studies as a predictor of arrhythmic events, and a 5% or 10% decrease of LVEF is associated with high arrhythmic risk [22].

Furthermore, Perry et al. demonstrated that in patients with moderate and severe decreased LVEF, mechanical dispersion ≥ 75 ms is related to VA, with a 9-fold increase in risk of VA events [35]. In a prospective study, patients with DCM and arrhythmic events had reduced LVEF and GLS, while mechanical dispersion predicted VA independently of LVEF [36].

## 4. Ecocardiography as an Adjunctive Tool for VA Ablation

Echocardiography may also offer specific relevant information prior to the ablation procedure. It is important to diagnose pericardial fluid prior to or during catheter ablation procedure. The presence of a cardiac thrombus should also be diagnosed before VA ablation. Traditionally, TTE was the main imaging tool to diagnose thrombosis, however recently CMR and cardiac CT are frequently used [6]. The guidelines suggest that although LV endocardial ablation can be performed in patients with a laminated thrombus, the presence of a mobile thrombus represents a contraindication [37]. In selected patients with mobile thrombi, ablation with epicardial access only has been demonstrated to be a good alternative [38].

Echocardiography has little contribuition in deciding the access route before catheter ablation. When femoral access is required, ultrasound-guided femoral arterial and venous access has been widely implemented in electrophysiological procedures in an effort to reduce vascular complications [39]. Sharma et al. reported the rate of major vascular access complications in patients undergoing VA ablation, which was 8.9% in the conventional group and 0% in the ultrasound-guided group [40]. Epicardial access is associated with higher complication rates and is not usually performed as the first line approach [6]. The decision depends on the underlying etiology and VT ECG characteristics. Late gadolinium enhancement CMR defines the location of epicardial or intramural scar and thus is more useful to establish the access route [6]. Imaging with cardiac CT or CMR can be used to assess the anatomic relationship between the pericardial space and the surrounding structures and this may limit the damage related to epicardial access [6].

Intracardiac echocardiography (ICE) generates 3-dimensional ventricular reconstruction that can be integrated in real-time with electroanatomic mapping. ICE provides real-time imaging of ventricular anatomy, catheter contact, and facilitates catheter manipulation during ablation [41].

In patients with scar-related VT, ICE may identify the thinned, akinetic myocardium and therefore it may localize the scar. There are studies which found a good correlation between scar area identified by ICE and that defined by EAM [42]. Furthermore, based on scar echo density, scar core has been differentiated from scar border zone as defined by EAM. ICE scar description has been validated against LGE-CMR and MDCT, but the technique is less effective [42]. In nonischemic cardiomyopathy, ICE can also detect the presence of midmyocardial and epicardial scar, which correlates with unipolar endocardial voltage abnormalities and epicardial bipolar voltage abnormalities [43].

LV access for endocardial mapping and VA ablation can be achieved either by retrograde transaortic or by antegrade transseptal approach. ICE has an important role in gaining safe left atrial access during transseptal puncture since it enables real-time visualization of the sheath-needle assembly, the fossa tenting and the puncturing the interatrial septum [41]. The technique is particularly valuable for VAs arising from sites with complex anatomy, such as periaortic VT and papillary muscle VT + RV free wall. ICE appears to be the most suitable imaging method for assessment of the exact location of the focus on papillary muscles in association with activation mapping [44].

ICE also provides early recognition of procedural complications, such as pericardial effusion or thrombus formation on sheaths and catheters. The presence of clots in the LV apex, especially in the setting of anteroapical aneurysms, can be evaluated with ICE to avoid catheter manipulation and prevent embolism [43]. Additional benefits are excellent patient tolerance and lack of need for general anesthesia or a second operator. For these reasons, ICE has largely replaced transesophageal echocardiography as ideal imaging modality for guiding catheter ablation of VA [43].

Futhermore, ICE facilitates significant reduction of radiation exposure during catheter ablation [45]. Zero fluoroscopy VT ablation is feasible using a combination of electroanatomic systems and ICE and achieving high acute success and low recurrence rates [41].

## 5. Conclusions

Conventional echocardiography has an established role in the management of patients suffering from ventricular arrhythmias, while novel echocardiographic techniques such as LV strain measurement and mechanical dispersion assessment upgrade the diagnostic and prognostic role of the technique. Additionally, intracardiac echocardiography may have an integrative role in real-time assessment of LV anatomy during VA ablation and early recognition of procedural complications.

## Figures and Tables

**Table 1:** Echocardiographic criteria for the diagnosis of cardiomyopathy.

Cardiomyopathy	Echocardiographic Parameters
HCM [7,8,9]	- LV maximal wall thickness > 15 mm * - Ratio of septal and posterior wall thickness ratio > 1.3 **
ARVC [10]	- Major criteria: Regional RV akinesia, dyskinesia, or aneurysm and one of the following: PLAX RVOT ≥ 32 mm (corrected for body size [PLAX/BSA] ≥ 19 mm/m^2^) PSAX RVOT ≥ 36 mm (corrected for body size [PSAX/BSA] ≥ 21 mm/m^2^) FAC ≤ 33% - Minor criteria: Regional RV akinesia or dyskinesia and one of the following: PLAX RVOT ≥ 29 to <32 mm (corrected for body size [PLAX/BSA] ≥16 to <19 mm/m^2^) PSAX RVOT ≥ 32 to <36 mm (corrected for body size [PSAX/BSA] ≥18 to <21 mm/m^2^) FAC > 33% to ≤40%
NCCM [11]	- Presence of numerous trabeculations and recesses: most proeminent at LV apex and least proeminent at the level of mitral valve - Ratio of thickness of NC and C myocardial layers at the site of maximal WT averages around 3.5 (2–3.5) - >3 trabeculations protrunding from the LV apex to the papillary muscles - Maximum linear length of NC-C myocardium - Planimetry of NC area on A4C
DCM [12]	- LVIDd >112% (2 s.d.) corrected for age and BSA **** - LVEF < 45% - LVFS < 25% - Mitral regurgitation - SI < 1.5 - Septal flash and apical rocking - Presence of dysynchrony indices

HCM—hypertrophic cardiomyopathy, ARVC—arrhythmogenic right ventricular cardiomyopathy, NCCM—noncompaction cardiomyopathy, DCM—dilated cardiomyopathy, SAM—systolic anterior motion, LVOT—left ventricular outflow tract, LV—left ventricle, RV—right ventricle, PLAX—parasternal long-axis view; RVOT—right ventricular outflow tract; BSA—body surface area; PSAX—parasternal short-axis FAC—fractional area change; NC—noncompacted, C—compacted, A4C—apical four chambers; LVIDd—Left ventricular internal dimension in diastole, LVEF—left ventricular ejection fraction, LVFS—left ventricular fractional shortening, SI—sphericity index; * > 13 mm in HCM relatives, ** > 1.5 in hypertensive patients, **** > 117% (2 s.d. plus 5%) increases the specificity and may be useful in familial screening.

**Table 2:** Etiology of VA.

VA in Structural Heart Disease	VA in Structurally Normal Heart
IHD pARVC HCM DCM NCCM Valvular heart disease (i.e., aortic stenosis, mitral valve prolapse) Congenital heart disease	Outflow tract VT Fascicular VT Interfascicular reentrant VT (Belhassen Tachycardia) Papillary muscle VA Channelopathies: - Long QT syndrome - Short QT syndrome - Brugada syndrome - Catecholaminergic polymorphic VT - Early repolarization syndrom

IHD—ischemic heart disease, ARVC—arrhythmogenic right ventricular cardiomyopathy, HCM—hypertrophic cardiomyopathy, DCM—dilated cardiomyopathy, NCCM—non compaction cardiomyopathy, VA—ventricular arrythmia, VT—ventricular tachycardia.

**Table 3:** Echocardiographic arrhythmogenic risk stratification in structural heart disease.

Cardiomyopathy	Echocardiographic Parameters
HCM [2,9,19]	LV wall thickness ≥ 30 mm LV systolic dysfunction LV apical aneurysm LA size LVOT gradient LV GLS Mechanical dispersion
ARVC [2]	Regional RV akinesia, dyskinesia or aneurysm RV diameter RV mechanical dispersion
NCM [20,21]	Ventricular dysfunction Hypoplasia of the compacted layer of the LV wall
DCM [22]	LV dysfunction/dilatation LV GLS LA size
ICM [2]	LV systolic dysfunction GWMSI RWT LV GLS/RLS Mechanical dispersion

ARVC—arrhythmogenic right ventricular cardiomyopathy, HCM—hypertrophic cardiomyopathy, DCM—dilated cardiomyopathy, NCCM—non compaction cardiomyopathy, LV—left ventricle, LVOT—left ventricular outflow tract, LA—left atrium, LGE—late gadolinium enhacement, RV—right ventricle, ICM—ischemic cardiomyopathy, GWMSI—global wall motion score index, RWT—relative wall thickness, GLS—global longitudinal strain.

## Data Availability

Not applicable.
